# Inhibition profile of trifludimoxazin towards PPO2 target site mutations

**DOI:** 10.1002/ps.7216

**Published:** 2022-10-17

**Authors:** Aimone Porri, Michael Betz, Kathryn Seebruck, Michael Knapp, Philipp Johnen, Matthias Witschel, Raphael Aponte, Rex Liebl, Patrick J. Tranel, Jens Lerchl

**Affiliations:** ^1^ BASF SE Ludwigshafen am Rhein Germany; ^2^ Department of Crop Sciences University of Illinois at Urbana‐Champaign College of Agricultural, Consumer and Environmental Sciences Urbana Illinois USA; ^3^ BASF Corporation Research Triangle Park North Carolina USA

**Keywords:** efficacy, herbicide, herbicide resistance, mutations, PPO, target site, trifludimoxazin

## Abstract

**BACKGROUND:**

Target site resistance to herbicides that inhibit protoporphyrinogen IX oxidase (PPO; EC 1.3.3.4) has been described mainly in broadleaf weeds based on mutations in the gene designated protoporphyrinogen oxidase 2 (PPO2) and in one monocot weed species in protoporphyrinogen oxidase 1 (PPO1). To control PPO target site resistant weeds in future it is important to design new PPO‐inhibiting herbicides that can control problematic weeds expressing mutant PPO enzymes. In this study, we assessed the efficacy of a new triazinone‐type inhibitor, trifludimoxazin, to inhibit PPO2 enzymes carrying target site mutations in comparison with three widely used PPO‐inhibiting herbicides.

**RESULTS:**

Mutated *Amaranthus* spp. PPO2 enzymes were expressed in *Escherichia coli*, purified and measured biochemically for activity and inhibition kinetics, and used for complementation experiments in an *E. coli hemG* mutant that lacks the corresponding microbial PPO gene function. In addition, we used ectopic expression in *Arabidopsis* and structural PPO protein modeling to support the enzyme inhibition study. The generated data strongly suggest that trifludimoxazin is a strong inhibitor both at the enzyme level and in transgenics *Arabidopsis* ectopically expressing PPO2 target site mutations.

**CONCLUSION:**

Trifludimoxazin is a potent PPO‐inhibiting herbicide that inhibits various PPO2 enzymes carrying target site mutations and could be used as a chemical‐based control strategy to mitigate the widespread occurrence of PPO target site resistance as well as weeds that have evolved resistance to other herbicide mode of actions. © 2022 BASF SE and The Authors. *Pest Management Science* published by John Wiley & Sons Ltd on behalf of Society of Chemical Industry.

## Graphical abstract

Protoporphyrinogen IX oxidase (PPO)‐inhibiting herbicide resistance is mainly endowed by target site mutations. *Amaranthus* plants may contain more than one PPO target site mutation. Trifludimoxazin is effective in controlling PPO enzymes carrying double mutations.

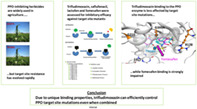

## 1 INTRODUCTION

Besides changes in regulatory requirements, herbicide resistance in agronomically important weeds is the most important driver of new herbicide development. Theoretically, hundreds of targets for herbicides that affect the growth and development of plants exist, yet the number of targets of commercial relevance for herbicide products is limited, as shown in Herbicide Resistance Action Committee (HRAC’s) mode of action and chemistry overview poster.^1^ As a complement to investigating new targets and new chemical motifs, researchers explore known chemical motifs by using molecular variations and combinations of chemical scaffolds for a known target.^2^, ^3^, ^4^, ^5^ One prominent example is protoporphyrinogen IX oxidase (PPO), the last shared enzyme of chlorophyll and heme biosynthesis in tetrapyrrole biosynthesis.

PPO inhibitors impair the PPO enzyme,^6^ which catalyzes conversion of protoporphyrinogen IX to protoporphyrin IX. A recent review summarizes all the steps involved in tetrapyrrole biosynthesis and some aspects of its regulation.^7^ The localization of the two PPO enzyme isoforms and specific sequence features have been summarized and discussed recently.^8^ Two types of PPO are found in tobacco, one in the chloroplasts and the other exclusive to the mitochondria,^9^ suggesting that the isoforms have independent roles in heme and chlorophyll synthesis.

Upon PPO enzyme inhibition, the PPO substrate protoporphyrinogen is exported outside the chloroplast and mitochondria, and strongly accumulates in the cytoplasm where nonspecific cytoplasmic oxidases convert it to protoporphyrin.^9^ Protoporphyrin ultimately reacts after exposure to sunlight, generating reactive oxygen species (oxygen singlet), which lead to lipid peroxidation and subsequent plant death.^10^ This is a unique, fast‐acting and light‐dependent mode of action.

The PPO enzyme is a particularly good target with respect to druggability. Structurally diverse chemistry classes are still being identified, allowing the synthesis of many new inhibitors.^2^, ^3^, ^4^ The first PPO inhibitor, nitrofen, was introduced onto the market in 1964. Despite more than half a century of chemical synthesis leading to thousands of synthesized molecules, PPO active ingredients with new characteristics can still be found. One of the latest PPO inhibitors to reach the market was tiafenacil in 2020.^11^

Commercially used PPO active ingredients applied in this study (saflufenacil, lactofen, fomesafen and the new active ingredient trifludimoxazin) show little selectivity, with strong and fast control of broadleaf weeds and lesser control of grasses. The difference in sensitivity between monocots and dicots was described early on as being caused by physiological differences.^12^ However, some PPO‐type herbicides such as oxadiazon and pyraclonil are used in turfgrass/rice to combat key grass weeds. In the weed *Eleusine indica*, a PPO1 target site mutation was found to mediate resistance specifically to oxadiazon.^13^ Herbicidal activity on grasses indicates that other factors such as the physicochemical parameters of the active ingredients, potentially including formulation of the herbicide, or intrinsic ADME parameters (absorption, distribution, metabolism and excretion) may also be important for weeds.^14^ So far, no significant differential target site activity on PPO enzymes from grasses or broadleaf weeds has been demonstrated.

During the past 15 years, PPO inhibitors have gained importance in the control of weeds resistant to glyphosate and ALS (acetolactate synthase) inhibitors.^15^ With this increasing use comes an increase in selection for PPO target site resistance among dicot weeds and grasses alike. Also, PPO‐tolerant traits in crops are likely to be released in the coming years^16^ and might be engineered using mutated resistant PPO2 plant enzyme^17^ or a bacterial PPO to a high level of herbicide tolerance in soybean, corn, canola and cotton,^18^ and this is expected to increase selection pressure. As PPO inhibitors use increases, it is important to monitor the development of herbicide resistance in the field and the impact of the mutations found.

Nontarget site resistance has been reported for fomesafen^19^ and carfentrazone‐ethyl including cross resistance to different PPO inhibitors (flumioxazin, acifluorfen and saflufenacil).^20^ Target site resistance to PPO‐inhibiting herbicides is mainly attributed to target site mutations on the PPO2 isoform, other than the single case in *E. indica* mentioned above. One PPO2 target site mutation has been identified in *A. artemisiifolia*, *A. tuberculatus* and *A. palmeri* as R128G, R128M, R128L (or R98L in *A. artemisiifolia*).^21^ These R128 substitutions cause loss of the salt bridge interaction between PPO2 and the ligand, reducing herbicide–target affinity. In addition, through modeling prediction,^22^ it was shown that R128G, M and L substitutions cause enlargement of the PPO2 active site, which becomes prone to a solvation effect, further reducing ligand–target affinity. A second mutation, a codon deletion resulting in loss of a glycine (ΔG210), in *A. tuberculatus* and *A. palmeri* was also shown to confer resistance to several PPO‐inhibiting herbicides including lactofen and fomesafen.^23^ In general, codon deletions are much less frequent than single nucleotide substitutions, with a frequency of approximately 10^−18^ compared with approximately 10^−9^, respectively^24^ However, the G210 codon deletion occurred in a genomic region with short simple sequence repeats. This simple sequence repeat microsatellite provides an environment in which a codon could be deleted through the slippage of DNA polymerase.^24^ Finally, a third target site mutation in Palmer amaranth, known as G399A (or G398 in *A. tuberculatus*), was reported to confer a high level of resistance to PPO inhibitors.^25^ Recent publications described the co‐occurrence of diverse PPO mutations in *A. tuberculatus* and/or *A. palmeri* populations collected in several US states.^22^, ^26^ Results show several predominant mutations, as described previously (ΔG210, R128G, and G399A),^25^ as well as new mutations and combinations thereof.^22^

In this study, we show the resistance profile of various *Amaranthus* PPO2 double target site mutants toward trifludimoxazin, and the resistance profiles are supported by analysis of major binding properties and in silico substrate binding to mutants.

## 2 MATERIAL AND METHODS

### 2.1 Evaluation of mutant PPO enzyme activity

Complete description of the expression and purification of *A. tuberculatus*, PPO2 variant proteins and the enzymatic assay to determine protein activity and median inhibitory concentration (IC_50_) is provided in Rangani et al.^25^

### 2.2 Molecular modeling for R128G, ΔG210 and G399A PPO enzymes

To investigate the effect of mutations on trifludimoxazin binding, we started with in‐house PPO2 structures co‐crystalized with trifludimoxazin as the basis for modeling. The crystal structure of the wild‐type, and of the ΔG210 variant, is available. Other PPO2 co‐crystal structures were used as a basis to model other herbicides, for example fomesafen into the binding site. After superposition on crystallographically observed binding modes, local minimization of the herbicides was performed. To examine the effect of the mutations G399A and R128G on ligand binding, the wild‐type crystal structure was modified by virtually mutating G399 and R128 to alanine and glycine, respectively. Local minimization of the complex was performed after introducing these mutations. For molecular modeling, the program MOE was used (Chemical Computing Group).

### 2.3 *Arabidopsis* transgenics growth and herbicide treatment

#### 2.3.1 Plant material and growth conditions

*Arabidopsis thaliana* seeds (stock MC24, from the Max Planck Institute for Molecular Plant Physiology at Golm) were sown in a substrate composed of GS90 soil + 5% sand. Seeds were subjected to stratification for 5 days at 4°C, followed by a short‐day growth period of 10 days (10:14 h day/night intervals at 20/18 ± 1°C, and approximately 120 μmol PAR (photosynthetically active ratio). After that, plants were transplanted into 8 × 8 cm pots filled with GS90 soil and cultivated under the same conditions for 14 days, under long‐day growth conditions (16:8 h day/night at 20/18 ± 1°C, approximately 200 μmol PAR) and maintained until seed harvest. Plants were fertilized with 0.3% Hakaphos Blau (15–10–15 NPK) twice a week until flowering. Relative humidity was not controlled, but kept between 40% and 70% during all growth stages, except during stratification.

#### 2.3.2 Transgene preparation

To prepare the transgene, wild‐type and mutant variants of *ppx‐2 L* from *A*. *tuberculatus* were inserted into a RTP6557 transformation vector, which was then inserted into *Agrobacterium tumefaciens* strain C58C1pMP90. The gene insert also included an acetolactate synthase herbicide‐resistance trait as a selectable marker to identify transformed *Arabidopsis* seedlings. This would ensure that plants eventually tested for resistance to PPO‐inhibiting herbicides all expressed the transgene.

#### 2.3.3 Bacterial culture and dipping medium

The *Agrobacterium* culture containing the plasmid was prepared a day before dipping by inoculating 1 ml of glycerol stock into 250 ml of YEB medium (1 g L^−1^ yeast, 5 g L^−1^ beef extract, 5 g L^−1^ peptone, 5 g L^−1^ sucrose, 0.49 g L^−1^ MgSO_4_ 7H_2_O) plus the appropriate antibiotic. Bacteria were cultured for 12 h at 28°C with continuous agitation at 150 rpm. The next day, after adjusting the *Agrobacterium* culture density to OD_600_ = 1.0 (with YEB medium), the culture was collected by centrifugation at 1600 g for 10 min and resuspended in 150 ml of infiltration medium composed of 2.2 g L^−1^ Murashige & Skoog (MS) medium, 50 g L^−1^ sucrose, 0.5 g L^−1^ MES hydrate, 10 μl L^−1^ BAP (benzylaminopurin, 1 mg ml^−1^). The pH was then adjusted to 5.7–5.8.

#### 2.3.4 Agrobacterium‐*mediated transformation of* Arabidopsis thaliana MC24 *by floral dip*

Plants with immature floral buds were dipped in the bacterial suspension for 10 s after adding 75 μl of Silwet‐L77 per 150 ml of infiltration medium to a jar. After dipping, plants were kept overnight in a cabinet under high humidity and low light intensity and were grown under long‐day conditions until maturity. When siliques turned yellow, plants were placed in paper bags to collect the seeds. T1 seeds were then transferred to falcon tubes and stored at 4°C.

#### 2.3.5 Selection of putative transformants with Imazamox and herbicide treatment

After 14 or more days of storage at 4°C, T1 seeds were sown to select putative transgenic *Arabidopsis* plants. Sowing and stratification were performed as described previously.^27^ Seeds were then treated with a 20 ppm imazamox (technical grade) solution and cultivated under short‐day growth conditions for 12–14 days, when resistant seedlings (four‐leaf stage) were transplanted into 6 × 6 cm pots filled with GS90 soil and grown for another 10 days. One day prior to herbicide application, growth conditions were set to ‘long‐day’ and maintained throughout the duration of the test. Herbicide treatments consisted of three concentrations of saflufenacil [Sharpen (BASF Corp.) at 10, 25 and 50 g active ingredient (a.i.) ha^−1^] and three concentrations of trifludimoxazin [Vulcarus (BASF Corp.) at 3.35, 6.75 and 12.5 g a.i. ha^−1^] foliar‐applied when plants reached the ten‐leaf stage, using a spray chamber calibrated to deliver 375 L ha^−1^ of spray solution. Herbicide efficacy was visually assessed after 7 days from herbicide treatments. Herbicides spray solution and empty control contain the adjuvant DASH HC (DASH) containing 349 g/l oil (fatty acid esters) and 209 g/l alkoxylated alcohols‐phosphate esters, reference ID no. 30059102, BASF.

### 2.4 HemG growth assay

The PPO2 R128(X) ΔG210 mutants of interest were cloned in the pET21 plasmid (ampicillin selection). The SAS38X cells were transformed by electroporation and plated on Luria–Bertani ampicillin agar plates. Colonies were visible after 48 h at 37°C. An overnight culture picked from a single colony was diluted with Luria–Bertani ampicillin for a starting OD_600_ of 0.05 in the assay. The herbicide starting concentration in the assay was 10^−5^ m with ten further dilutions 1:5 in 60% dimethyl sulfoxide. Ten microliters of herbicide was added to 190 μl of prepared cells to give a total assay volume of 200 μl. The assay was prepared in a clear and sterile 96‐well flat bottom plate with a lid. Absorbance was measured every 30 min at 600 nm (OD_600_) for 24 h at 37°C with shaking.

#### 2.4.1 Data analysis and evaluation

The maximum slope was determined using at least eight measuring points (*V*_max_). The *V*_max_ value, the maximum enzyme velocity under saturated substrate condition, of the positive control (cells only with dimethyl sulfoxide) is equivalent to 0% inhibition. Calculation of the percent inhibition values for each herbicide in all ten dilutions determined the IC_50._value

## 3 RESULTS

### 3.1 Trifludimoxazin major binding properties

**FIGURE 1:**
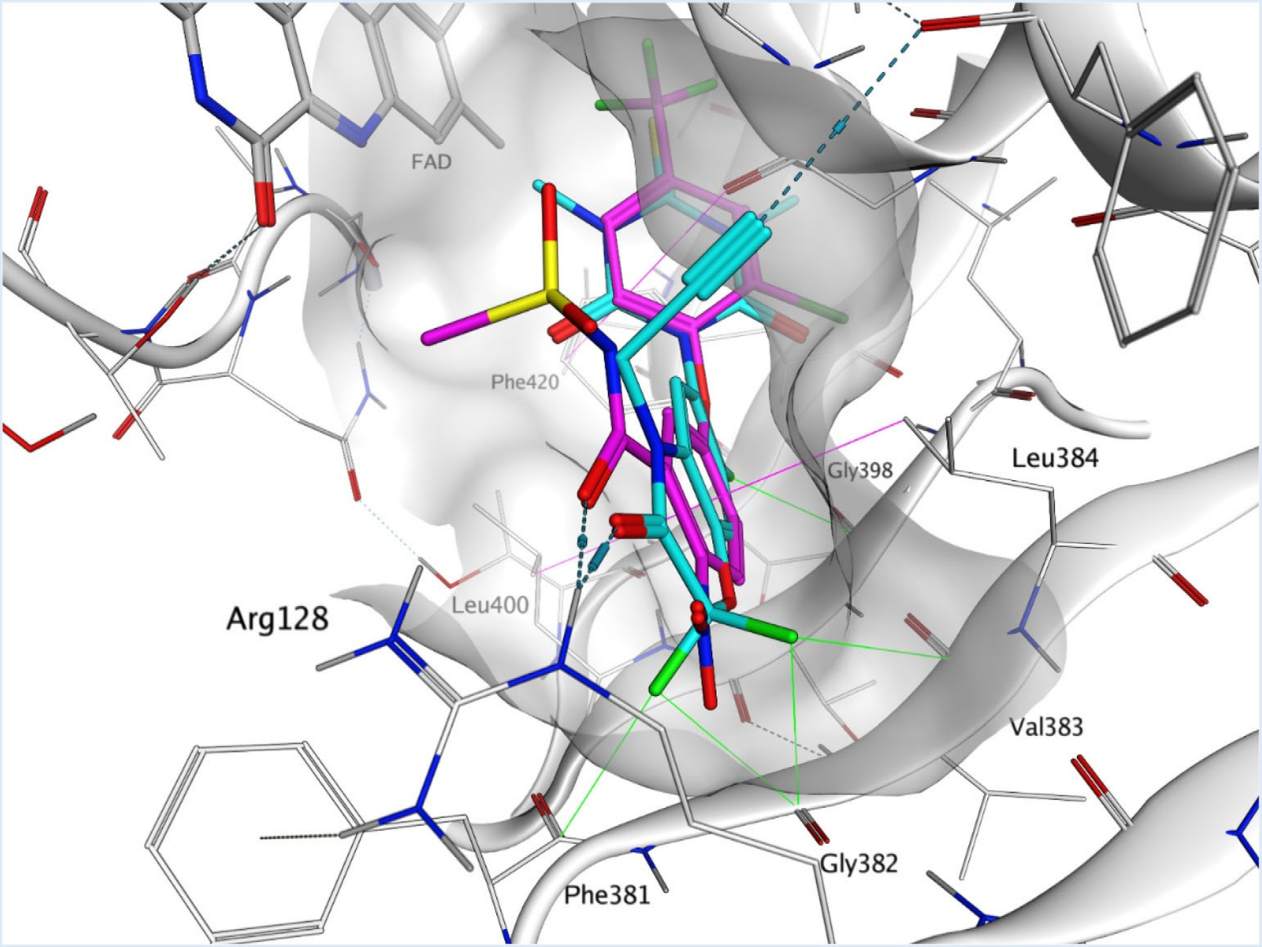
Binding mode of trifludimoxazin and binding pose fomesafen in *Amaranthus tuberculatus* protoporphyrinogen oxidase 2 (PPO2) wild‐type. The protein crystal structure of PPO2 co‐crystalized with trifludimoxazin is shown in white. Secondary structural elements are shown in cartoon style. A white surface shows the shape of the binding site. Trifludimoxazin is shown in stick representation with carbon in cyan, the binding mode of the co‐factor flavin adenine dinucleotide in gray and the modeled binding pose of fomesafen in magenta. Oxygen is shown in red, nitrogen in blue, sulfur in yellow and fluorine in green. The binding of PPO2 inhibitors is stabilized by pi‐stacking effects of the ring systems (magenta lines). The triazindionthione head group with the sulfur and two methyl groups fits very well in a hydrophobic environment and is covered between Phe420 and the tip of an α‐helical structure. The heterocyclic benzoxazinone exhibits many favoring van der Waals interactions with the β‐sheet elements shaping the binding site of protoporphyrinogen IX oxidase. The hydrophobic amino acids Leu384 and Leu400 interact favorably with the pi‐electron system. The propargyl group anchors in a small hydrophobic pocket, establishing a bond with a carbonyl via the polarized hydrogen. Arg128 can establish a charge‐assisted hydrogen bond upon binding. In particular, the CF2 group, with its multipolar interactions (green lines) to the carbonyl oxygens of Phe381, Gly382 and Val383 protein backbone, but also to Gly398, is key for affinity. Mutations at the position of Phe381 or Val383 would not change the interaction between the fluorine atoms to the backbone because the altered side chains would point away from trifludimoxazin. Any mutation of Gly382, the middle amino acid, generally results in an inactive protein.

An overview on the major herbicidal properties of trifludimoxazin has been published elsewhere.^28^ The aim of this report is to concentrate on the binding properties of this herbicide. Like other PPO inhibitors, trifludimoxazin acts by blocking the function of the plant enzymes protoporphyrinogen oxidase 1 and 2 (PPO1 and PPO2). Compared with other PPO herbicides, such as fomesafen or lactofen, trifludimoxazin binds with stronger affinity to the PPO2 active site through additional interactions with the enzyme beta backbone (Figure 1). A difluoro group was added to the precursor of trifludimoxazin by structural‐based design (by docking the herbicide to the PPO2 active site) to create a unique binding profile. Because of multipolar interactions, the difluoro group (Figure 1) creates high affinity with the backbone atoms Phe381, Gly382 and Val383 of PPO2, which ensures tight binding to the target.

### 3.2 Resistance profile of recombinant R128(X) ΔG210 double‐ and G399A single‐mutant enzyme variants towards trifludimoxazin

To assess the inhibition potency of trifludimoxazin towards these double‐ and single‐mutant PPO2 enzymes, an in vitro inhibition assay was performed. PPO2 double‐mutant variants were expressed in *Escherichia coli*, purified and mixed with a concentration range of four PPO inhibitors: trifludimoxazin, lactofen, fomesafen and saflufenacil. The latter three were used as benchmarks. Following enzyme activity measurements of PPO2 variants at different herbicide concentrations, enzyme inhibition kinetics (IC_50_) were calculated. IC_50_ is the concentration of a given herbicide that causes 50% in vitro protein inhibition and reflects the herbicide inhibition potency. Therefore, high IC_50_ values indicate a poor inhibition effect, whereas low IC_50_ values indicate strong inhibitory potency (Tables 1, 2, 3). The IC_50_ of trifludimoxazin for the G399A enzyme was lower than for fomesafen, lactofen and saflufenacil indicating a greater inhibitory potency (Table 3). R128(X) G399A were all inactive and below the detection limit of the assay, thus the IC_50_ values could not be determined.

**TABLE 1:** IC_50_ values of protoporphyrinogen oxidase 2 double‐mutant variants. IC_50_ is the amount of a given herbicide that inhibits 50% of the recombinant protein activity in vitro

	IC_50_ (m)			
Mutant variant	Trifludimoxazin	Saflufenacil	Lactofen	Fomesafen
Wild‐type	2.45 × 10^−10^	1.89 × 10^−9^	4.15 × 10^−10^	7.13 × 10^−9^
ΔG210	1.83 × 10^−9^	2.04 × 10^−6^	5.44 × 10^−7^	>1 × 10^−5^
R128A ΔG210	3.30 × 10^−8^	>1 × 10^−5^	>1E × 10^−5^	>1 × 10^−5^
R128C ΔG210	3.11 × 10^−8^	>1 × 10^−5^	2.15 × 10^−6^	>1 × 10^−5^
R128D ΔG210	Inactive	Inactive	Inactive	Inactive
R128F ΔG210	Inactive	Inactive	Inactive	Inactive
R128G ΔG210	Inactive	Inactive	Inactive	Inactive
R128H ΔG210	3.02 × 10^−8^	>1 × 10^−5^	1.59 × 10^−6^	>1 × 10^−5^
R128I ΔG210	9.93 × 10^−8^	>1 × 10^−5^	1.95 × 10^−6^	>1 × 10^−5^
R128E ΔG210	8.61 × 10^−9^	>1 × 10^−5^	6.49 × 10^−7^	>1 × 10^−5^
R128K ΔG210	Inactive	Inactive	Inactive	Inactive
R128L ΔG210	5.68 × 10^−9^	>1 × 10^−5^	6.49 × 10^−7^	>1 × 10^−5^
R128M ΔG210	1.82 × 10^−7^	>1 × 10^−5^	>1 × 10^−5^	>1 × 10^−5^
R128N ΔG210	1.75 × 10^−7^	>1 × 10^−5^	>1 × 10^−5^	>1 × 10^−5^
R128Q ΔG210	1.45 × 10^−8^	>1 × 10^−5^	1.75 × 10^−6^	>1 × 10^−5^
R128T ΔG210	7.48 × 10^−8^	>1 × 10^−5^	3.92 × 10^−6^	>1 × 10^−5^
R128Y ΔG210	Inactive	Inactive	Inactive	Inactive
R128P ΔG210	Inactive	Inactive	Inactive	Inactive
R128S ΔG210	Inactive	Inactive	Inactive	Inactive
R128V ΔG210	Inactive	Inactive	Inactive	Inactive
R128W ΔG210	Inactive	Inactive	Inactive	Inactive

*Note*: Measurements were performed in three technical replicates.

**TABLE 2:** Resistance factors of the protoporphyrinogen oxidase 2 double‐mutant variants

	Resistance factor			
Mutant variant	Trifludimoxazin	Saflufenacil	Lactofen	Fomesafen
Wild‐type	1	1	1	1
ΔG210	7	1 079	1 311	>1 403
R128A ΔG210	135	>5 291	>24 096	>1 403
R128C ΔG210	127	>5 291	5 181	>1 403
R128H ΔG210	123	>5 291	3 831	>1 403
R128I ΔG210	405	>5 291	4 699	>1 403
R128E ΔG210	35	>5 291	1 564	>1 403
R128L ΔG210	23	>5 291	1 564	>1 403
R128M ΔG210	743	>5 291	>24 096	>1 403
R128N ΔG210	714	>5 291	>24 096	>1 403
R128Q ΔG210	59	>5 291	4 217	>1 403
R128T ΔG210	305	>5 291	9 446	>1 403

*Note*: Resistance factors were calculated by dividing the median inhibitory concentration (IC_50_) of the given variant by the IC_50_ of wild‐type protoporphyrinogen oxidase 2.

**TABLE 3:** IC_50_ values of protoporphyrinogen oxidase 2 double‐mutant variants. IC_50_ is defined as the amount of a given herbicide that inhibits 50% of the recombinant protein activity in vitro

	IC_50_ (m)			
Mutant variant	Trifludimoxazin	Saflufenacil	Lactofen	Fomesafen
Wild‐type	2.45 × 10^−10^	1.89 × 10^−9^	4.15 × 10^−10^	7.13 × 10^−9^
G399A	1.17 × 10^−9^	6.27 × 10^−7^	1.58 × 10^−6^	>1 × 10^−5^
∆G210 G399A	Inactive	Inactive	Inactive	Inactive
R128A G399A	Inactive	Inactive	Inactive	Inactive
R128C G399A	Inactive	Inactive	Inactive	Inactive
R128D G399A	Inactive	Inactive	Inactive	Inactive
R128F G399A	Inactive	Inactive	Inactive	Inactive
R128G G399A	Inactive	Inactive	Inactive	Inactive
R128H G399A	Inactive	Inactive	Inactive	Inactive
R128I G399A	Inactive	Inactive	Inactive	Inactive
R128E G399A	Inactive	Inactive	Inactive	Inactive
R128K G399A	Inactive	Inactive	Inactive	Inactive
R128L G399A	Inactive	Inactive	Inactive	Inactive
R128M G399A	Inactive	Inactive	Inactive	Inactive
R128N G399A	Inactive	Inactive	Inactive	Inactive
R128Q G399A	Inactive	Inactive	Inactive	Inactive
R128T G399A	Inactive	Inactive	Inactive	Inactive
R128Y G399A	Inactive	Inactive	Inactive	Inactive
R128P G399A	Inactive	Inactive	Inactive	Inactive
R128S G399A	Inactive	Inactive	Inactive	Inactive
R128V G399A	Inactive	Inactive	Inactive	Inactive
R128W G399A	Inactive	Inactive	Inactive	Inactive

*Note*: Measurements were performed in three technical replicates.

The IC_50_ values of lactofen for R128(X) ΔG210 double‐mutant enzymes ranged from 10^−7^ to 10^−6^ for R128C ΔG210, R128E ΔG210, R128H ΔG210, R128I ΔG210, R128L ΔG210, R128Q ΔG210 and R128T ΔG210. IC_50_ values greater than 10^−5^ (which was assessed as highly resistant) were found for R128A ΔG210, R128M ΔG210 and R128N ΔG210. The other nine variants were inactive or below the detection limit of the assay. The inhibition potency of saflufenacil and fomesafen towards R128(X) ΔG210 mutants was low (IC_50_ below 10^−5^ m) in most cases, indicating poor inhibition potency. Thus, lactofen appears to be a better inhibitor than saflufenacil and fomesafen in inhibiting PPO2 enzymes carrying R128(X) ΔG210 double mutations. R128D ΔG210, R128S ΔG210, R128Y ΔG210, R128F ΔG210, R128P ΔG210, R128W ΔG210, R128V ΔG210 and R128G ΔG210 are less likely to occur in planta because these double‐mutant enzymes were found to be inactive in vitro and are likely to impose a significant plant fitness cost. The IC_50_ values for trifludimoxazin were much lower than those found for saflufenacil, fomesafen and lactofen, and ranged from 10^−9^ to 10^−7^ m, showing that this inhibitor has a greater ability to inhibit R128(X) ΔG210 mutants at the enzyme level. To confirm this further, the plant response (ED_50_) values of lactofen, saflufenacil and trifludimoxazin were determined using an in vivo growth‐based system that utilizes the hemG *E. coli* strain complemented with *Amaranthus* PPO2. In agreement with the in vitro enzyme assay results, trifludimoxazin showed the highest inhibition potency against heterologously expressed R128(X) ΔG210 variants (Table 4). The *E. coli* hemG strain was not sensitive to fomesafen, probably because of impaired uptake or hydrolysis of the compound (data not shown).

**TABLE 4:** IC_50_ values of protoporphyrinogen oxidase 2 double‐mutant variants tested in *Escherichia coli* hemG system. IC_50_ is defined as the amount of a given herbicide that inhibits 50% of the *E. coli* hemG growth

	IC_50_ (m)		
Mutant variant	Trifludimoxazin	Saflufenacil	Lactofen
Wild‐type	9.27 × 10^−10^	3.19 × 10^−8^	6.24 × 10^−8^
ΔG210	1.18 × 10^−9^	2.12 × 10^−6^	3.57 × 10^−7^
R128A ΔG210	4.65 × 10^−8^	>1.00 × 10^−5^	>1.00 × 10^−5^
R128C ΔG210	4.65 × 10^−8^	>1.00 × 10^−5^	>1.00 × 10^−5^
R128H ΔG210	9.00 × 10^−8^	>1.00 × 10^−5^	>1.00 × 10^−5^
R128I ΔG210	8.29 × 10^−8^	>1.00 × 10^−5^	>1.00 × 10^−5^
R128E ΔG210	9.87 × 10^−9^	>1.00 × 10^−5^	2.89 × 10^−6^
R128L ΔG210	3.99 × 10^−8^	>1.00 × 10^−5^	>1.00 × 10^−5^
R128M ΔG210	6.74 × 10^−8^	>1.00 × 10^−5^	>1.00 × 10^−5^
R128N ΔG210	9.87 × 10^−9^	>1.00 × 10^−5^	>1.00 × 10^−5^
R128Q ΔG210	9.86 × 10^−9^	>1.00 × 10^−5^	>1.00 × 10^−5^
R128T ΔG210	6.75 × 10^−8^	>1.00 × 10^−5^	>1.00 × 10^−5^

Note: Measurements were performed in three technical replicates.

### 3.3 Likelihood of double‐mutant combination in PPO2

The likely occurrence of amino acid substitutions at the position 128 in PPO2 of *A. palmeri* and *A. tuberculatus* was predicted by in silico mutating each nucleotide of the triplet that encodes R128. Single mutation on the triplet is more likely to occur than double or triple mutations. R128 is encoded by an AGG codon in *A. palmeri* and AGA codon in *A. tuberculatu*s (Table 5). The in silico mutagenesis resulted in the following possible substitution: R128G, R128K, R128M, R128S and R128T for *A. palmeri*, and R128G, R128I, R128K, R128S and R128T for *A. tuberculatus* (Table 5). Thus, the above substitutions are more likely to evolve as a result of selective pressure imposed by PPO‐inhibiting herbicides and could occur in combination with ΔG210. The other substitutions require more than one nucleotide change and are therefore less likely to evolve.

**TABLE 5:** Effect of one single nucleotide change at the position R128 of protoporphyrinogen oxidase 2 in *Amaranthus palmeri* and *Amaranthus tuberculatus*

Amino acid	Codon(s)
*Amaranthus palmeri*	
Cysteine (C)	TGT TGC
Phenylalanine (F)	TTT TTC
Glycine (G)	GGT GGC GGA GGG
Histidine (H)	CAT CAC
Isoleucine (1)	ATT ATC ATA
Lysine (K)	AAA AAG
Leucine (L)	CTT CTC CTA CTG TTA TTG
Methionine (M)	ATG
Asparagine (N)	AAT AAC
Glutamine (Q)	CAA CAG
Serine (S)	TCT TCC TCA TCG AGT AGC
Threonine (T)	ACT ACC ACA ACG
Valine (V)	GTT GTC GTA GTG
Tyrosine (Y)	TAT TAC
*Amaranthus tuberculatus*	
Cysteine (C)	TGT TGC
Phenylalanine (F)	TTT TTC
Glycine (G)	GGT GGC GGA GGG
Histidine (H)	CAT CAC
Isoleucine (I)	ATT ATC ATA
Lysine (K)	AAA AAG
Leucine (L)	CTT CTC CTA CTG TTA TTG
Methionine (M)	ATG
Asparagine (N)	AAT AAC
Glutamine (Q)	CAA CAG
Serine (S)	TCT TCC TCA TCG AGT AGC
Threonine (T)	ACT ACC ACA ACG
Valine (V)	GTT GTC GTA GTG
Tyrosine (Y)	TAT TAC

*Note*: Possible R128 substitutions are shown in orange for *A. palmeri* and in blue for *A. tuberculatus*. R128 is encoded by a AGG triplet in *A. palmeri* and AGA in *A. Tuberculatus*.

### 3.4 R128(X) ΔG210 ectopic expression in transgenic *Arabidopsis*

**FIGURE 2:**
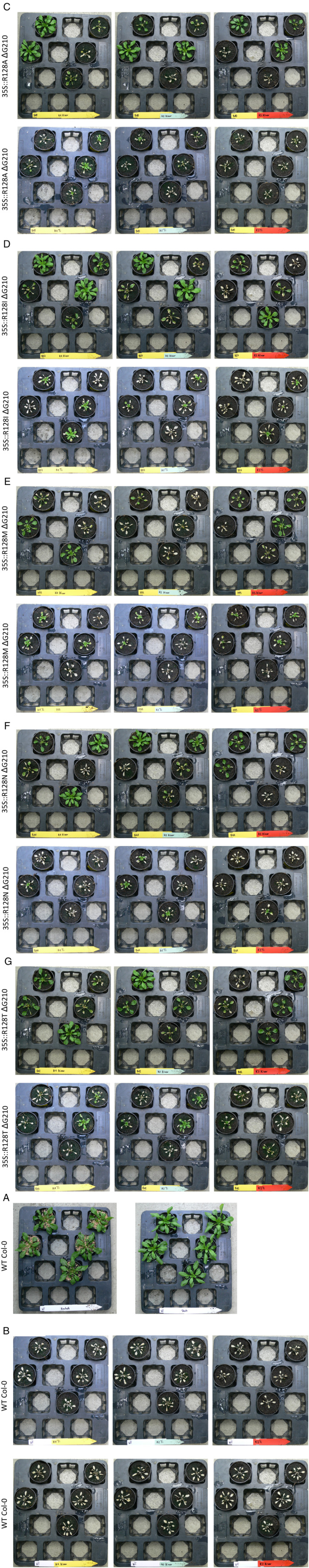
T1 35S::AMATU PPO2 R128(X) ∆G210 *Arabidopsis* lines sprayed with saflufenacil and trifludimoxazin. Pictures were taken 7 days after treatment. The five pots contain independent transgenic events (T1 plants, selected with Imazamox by confirming presence of resistance gene AHAS). (a) Untreated wild‐type (WT) *Arabidopsis* (left) and treatment with the adjuvant DASH (right). (b) WT *Arabidopsis* treated with 2×, 1× and 0.5× (right to left) saflufenacil (upper) or trifludimoxazin (lower). (c–g) T1 *Arabidopsis* transgenics treated with 2×, 1× and 0.5× saflufenacil (upper) or trifludimoxazin (lower). 1× trifludimoxazin = 12.5 g ha^−1^; 1× saflufenacil = 25 g ha^−1^.

To further confirm that trifludimoxazin has a greater efficacy against R128(X) ΔG210 target site mutations, the mutants were ectopically expressed in *Arabidopsis thaliana*, and the transgenic lines were treated with 2×, 1× and 0.5× the field dose concentrations of trifludimoxazin or saflufenacil (Figure 2). The R128(X) ΔG210 variants that showed the higher resistance factor in the inhibition studies toward trifludimoxazin (R128A ΔG210, R128I ΔG210, R128M ΔG210, R128N ΔG210 and R128T ΔG210) (Table 2) were employed in this experiment. Five independent T1 transgenic lines expressing PPO2 variants were tested. Based on visual assessment, two T1 lines expressing R128A ΔG210 showed significant resistance at each applied rate of saflufenacil, whereas trifludimoxazin reduced plant growth at 2×, 1× and 0.5× the rate of all five T1 plants. Likewise, R128I ΔG210 transgenics were more controlled by trifludimoxazin than saflufenacil at all the used rates. The growth of R128N ΔG210 and R128T ΔG210 transgenics line was also more reduced by trifludimoxazin than saflufenacil at each rate. The growth of R128M ΔG210 transgenics was reduced by both trifludimoxazin and saflufenacil at 1×, whereas few T1 plants showed persistent growth with saflufenacil at the 2× and 0.5× rate. Trifludimoxazin reduced the growth of R128M ΔG210 transgenics at the 2× and 1× rate to a greater extent than saflufenacil. The above experiments support the idea that trifludimoxazin provides greater growth reduction of PPO2 double target site mutations, in agreement with in vitro inhibition studies.

### 3.5 Molecular modeling for R128G, ΔG210 and G399A PPO enzyme

**FIGURE 3:**
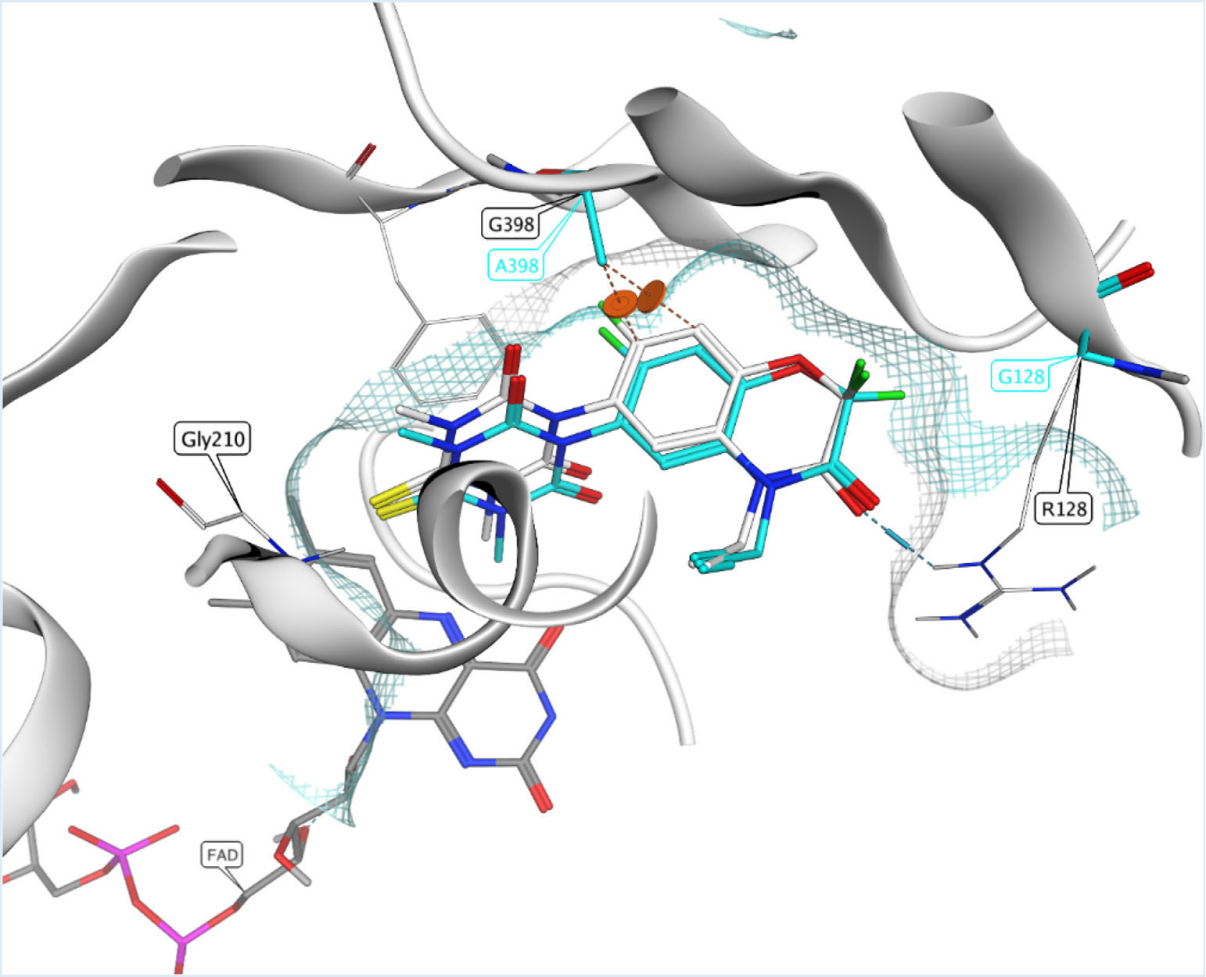
Trifludimoxazin binding in *Amaranthus tuberculatus* protoporphyrinogen oxidase 2 (PPO2) wild‐type with mutation sites. The protein crystal structure of PPO2 together with trifludimoxazin is shown in white. The binding mode of trifludimoxazin in the presence of target site mutations is shown in cyan. The β‐sheet wall (upper), and the α‐helical structure (middle) are depicted white, in cartoon style. The co‐factor flavin adenine dinucleotide is depicted by sticks with gray carbon color. Oxygen is shown in red, nitrogen in blue, sulfur in yellow and fluorine in green. The shape of the wild‐type binding site is highlighted by white hatching. R128G and G398A (cyan stick representation) reshaping the binding site are highlighted by blue hatching.

A detailed study of the binding mode of trifludimoxazin, for which internal protein crystal structures were created, reveals several features that explain the high affinity to plant PPO. Together with molecular modeling, it could be explained how trifludimoxazin inhibits the major PPO2 target site mutations including double‐mutant combinations. Trifludimoxazin binds in the same substrate‐binding pocket as protoporphyrinogen, right next to the co‐factor flavin adenine dinucleotide (FAD). The pocket is deeply buried in the protein. Beta‐sheet secondary structures form the posterior wall of the pocket, which is covered from above by an α‐helical structure and defined from below by the co‐factor (Figure 3). Trifludimoxazin binding is characterized by an excellent shape complementarity between the herbicide and the binding pocket, leading to many favorable van der Waals interactions. In addition, binding is stabilized by the pi‐stacking effects of both trifludimoxazin ring systems. The body of the molecule forms the fluorinated heterocyclic benzoxazinone, which is sandwiched in between hydrophobic amino acids and produces favorable interactions via its pi‐electron system. Fluorine atoms, in particular the CF2 group, are key for affinity on PPO with its multipolar interactions to the carbonyl oxygens of these β‐sheet elements.^28^ The exocyclic oxygen of benzoxazinone can form a salt bridge to the flanking arginine 128, the propargyl group anchors in a small hydrophobic pocket of the protein and interacts via the polarized hydrogen with a carbonyl of the α‐helical structure. Both features support binding to the protein; the 6‐thioxo‐1,3,5‐triazinane‐2,4‐dione head group is entrapped from two sides. It establishes pi‐stacking interactions with the underlying ring system of a phenylalanine and interacts itself with the tip of the α helix on top. Sulfur and methyl groups fit very well in the aliphatic region of the protein (Figure 3). Using molecular modeling, changes in shape of the binding site and protein–ligand interactions caused by the mutations were analyzed and interpreted. In the case of the ΔG210 variant, deletion of an entire amino acid in the α‐helical structure leads to a larger binding site compared with the wild‐type so that the head group loses some favorable hydrophobic interactions. Another effect of the widened pocket is that water molecules now enter more easily and thus compete more strongly with trifludimoxazin binding. These two effects explain the weakened affinity of the variant compared with the wild‐type. The R128G mutation on the opposing side has a similar effect on the integrity of the binding pocket. The mutation of arginine 128 to glycine leads, on the one hand, to the loss of a salt bridge between trifludimoxazin and the protein and, on the other hand, to the loss of the long side chain that previously covered the binding pocket. Here, the binding pocket also becomes much more accessible to the solvent. The G399A mutation occurs in the exact pathway in which PPO inhibitors bind to the β‐sheet wall. When glycine is mutated to any other amino acid, the side chain protrudes into the pocket, directly towards the inhibitors. In the case of alanine, the additional methyl group changes the shape of the binding pocket on the β‐sheet wall. This has devastating consequences for many known PPO inhibitors, resulting in a dramatic decrease in binding affinity because tight binding is no longer possible. However, the shape and size of trifludimoxazin allow the molecule to compensate for this change, so that good shape complementarity continues to exist and the unique CF2 interactions are maintained. Trifludimoxazin is much less sensitive to the mentioned mutations than other PPO herbicides. In particular, tight binding of the body to the β‐sheet wall, with its multipolar interactions through the CF2 group, is not affected by the known mutations.

## 4 DISCUSSION

PPO‐inhibiting herbicides are effective tools to control a broad spectrum of weeds, including those that have evolved resistance to glyphosate.^29^ In 2004, the first crystal structure of a plant PPO enzyme bound to an inhibitor was published.^30^ Analysis of this structure shows some important features of the binding site, such as the FAD, helix tip and extended β sheet.^28^, ^30^ The major interactions of the PPO enzyme with the inhibitor are based on interactions with amino acid side chains.^28^ Interactions with the β sheet are only minor, even though this structure encompasses the active site. Therefore, these interactions have been exploited to increase binding properties to fully inhibit PPO enzymes carrying target site mutations that often occur in the side chain and not in the backbone.^28^ This approach aimed to target polar interactions of the fluorine atoms of the inhibitor with the carbonyl groups of the PPO backbone and ultimately resulted in trifludimoxazin, a new potent PPO inhibitor with superb herbicidal properties.^28^ Addition of a difluoro group was showed to confer strong inhibition properties to trifludimoxazin. This compound showed a significant higher inhibition potency than a difluorinated‐flumioxazin like compound on the wild‐type PPO2 enzyme.^28^

Mutations on Phe381 and Val383 of PPO2 were predicted to not affect trifludimoxazin binding because their side chains are directed away from the binding site (Figure 1). Substitutions of Gly382 result in inactivation of PPO2. Therefore, variations at Phe381, Gly382 and Val383 residues are unlikely to lead to trifludimoxazin resistance. Because of multiple interactions with the protein backbone (Figure 1), trifludimoxazin binding is less affected by R128G, ΔG210 and G339A PPO2 target site mutations.^28^

Resistance to PPO‐inhibiting herbicides is largely due to mutations on the PPO1 and PPO2 genes. Understanding the implication of these mutations is necessary for maintaining the usefulness of PPO herbicides as tools for modern agriculture. As mentioned above, three main mutations in the PPO2 protein endow most of the resistance cases. These mutations are R128G, ΔG210 and G399A. It was reported that ΔG210 deletion causes the unravelling of the turn of the PPO2 helix, leading to 50% enlargement in the active site, which can now fit both the substrate and the inhibitor.^24^ By contrast,^22^ G210 deletion causes a change in the dynamic of the α helix, which becomes more flexible and allows water molecules (solvation) to enter the active site, thus reducing herbicide binding, which in general requires a hydrophobic environment. The principal difference observed between G399 and A399 is the additional methyl group (‐CH3) that creates close repulsive interaction with the central phenyl ring of PPO inhibitors generating repulsive electrostatic interactions that push the herbicide from the binding site.^25^

Recently, Porri *et al*.^27^ discussed the likelihood of a double PPO2 mutation on the same allele. Although R128G and ΔG210 can occur on the same allele, the presence of these mutations on both alleles is unlikely because of the fitness cost associated with this mutant combination.^27^ Nevertheless, other double‐mutant combinations, R128(X) ΔG210, may appear in the future if the imposed fitness cost is less severe. In silico mutagenesis showed that the following substitutions are possible based on one nucleotide change: R128G, R128K, R128M, R128S and R128T for *A. palmeri*; and R128G, R128I, R128K, R128S and R128T for *A. tuberculatus* (Table 5). Thus, the above substitutions are more likely to evolve as a result of selective pressure imposed by PPO‐inhibiting herbicides and could occur in combination with ΔG210 if sufficient protein activity is retained. The other substitutions require more than one nucleotide change and are therefore less likely to evolve. G399A in combination with ΔG210 or R128(X) resulted in inactive PPO2 enzymes (Table 3).

In this article, we show the inhibitory capability of trifludimoxazin toward PPO2 enzymes carrying target site mutations. At the PPO2 enzyme level, trifludimoxazin was shown to inhibit R128L, R128G R128M and ΔG210 to a greater extent than other competitor benchmarked products, including saflufenacil, butafenacil, lactofen and fomesafen.^28^ Our data indicate that trifludimoxazin can inhibit PPO2 carrying double‐mutant combinations to a greater extent than any of the other PPO herbicides tested. A unique mode of binding differentiates trifludimoxazin from all other known PPO herbicides. The growth of *Arabidopsis* transgenic plants expressing R128(X) ΔG210 double‐mutant enzyme variants was reduced more by trifludimoxazin than by saflufenacil (Fig. 2). It is important to note that although a greater reduction in the growth of T1 plants was observed for trifludimoxazin, this did not completely destroy the treated plants and some regrowth might be expected. This seems to be in agreement with previous work in which the authors showed some levels of tolerance of T1 transgenic line expressing R128A ΔG210 and R128L ΔG210 when treated with trifludimoxazin. This might be explained in part by the high level of PPO2 expression driven by the *35S*:: promoter, which would result in a greater level of tolerance than the endogenous PPO2 promoter.

*Arabidopsis* is commonly used to screen the effect of herbicide‐resistance genes,^31^ but it is important to note that its response to the herbicide treatments may differ slightly from *Amaranthus*. Therefore, controlling *Arabidopsis* plants at 1× and 2× the field rates of trifludimoxazin does not fully guarantee control of *Amaranthus* expressing PPO2 double mutations. However, all the above indications point out that trifludimoxazin has greater inhibition capability than the other PPO inhibitors tested towards PPO2 enzymes carrying target site mutations. This might be translated into greater biomass control of resistant weeds in the field.

At the enzyme level, trifludimoxazin exhibited a lower resistance factor when compared with saflufenacil, lactofen and fomesafen. This was also confirmed in an in vivo growth‐based assay that utilizes the *E. coli* mutant hemG for lactofen, trifludimoxazin and saflufenacil (Tables 1 and 4). Unfortunately, yeast HemG was not sensitive to fomesafen, which shows some limitation in the use of this in vivo system to assesses the inhibition potency of certain PPO herbicides. The modeling data also support the experimental results (Figure 3). At the protein level, R128(X) substitutions and ΔG210 are likely to change the dynamic of the active site and allow a solvation effect in which water can enter the active site, compromising the hydrophobic environment.^22^ The difluoro group, a unique feature of trifludimoxazin, assures strong interactions with the enzyme backbone and overcomes R128(X) substitution and ΔG210 mutations even when combined. Whether certain double‐mutant combinations are more likely to appear in the field will probably depend on the fitness cost that these double mutations impose on the PPO enzymes. Trifludimoxazin also inhibits the function of the PPO1 isoform to a greater extent than other commercially available PPO inhibitors.^28^ Trifludimoxazin also shows grass suppression for certain species including *Lolium rigidum*. PPO1 target site resistance was recently found in the grass *Eleusine indica*.^13^ An A212T substitution in the PPO1 isoform was suggested to confer resistance specifically to oxadiazon, and is the first example to date of PPO resistance endowed by the PPO1 isoform. Trifludimoxazin can fully inhibit the PPO1 enzyme carrying A212T mutation, suggesting that this herbicide may be opted to combat target site mutants in grass species as well.^13^ Trifludimoxazin can be applied in preplant burndown, but also shows relatively low mobility in the soil and residuality resulting in particularly potent activity in pre‐eVulcarmergence application, making it an effective weed control option at different cropping stages. Crop safety is achieved by sowing seeds at least 2 weeks after trifludimoxazin application, seed placement and crop ADME properties likely contribute to the selectivity as well.

Mitigating herbicide resistance often requires a diversified strategy that does not rely only on the use of chemicals. Although trifludimoxazin is probably the most potent PPO‐inhibiting herbicide on the market, sole reliance on this herbicide may impose strong selective pressure that promotes resistance. Therefore, use of trifludimoxazin should always be combined with other control strategies to preserve its efficacy in the long term and slow the evolution of resistance.

## 5 CONCLUSION

Compared with the other PPO inhibitors evaluated, trifludimoxazin provides greater inhibition of PPO2 enzyme with mutations related to herbicide resistance, even when they are combined. Consequently, trifludimoxazin has potential to combat existing weed populations with target site resistance to PPO inhibitors. Further molecular structure‐based design of PPO‐inhibiting herbicides is needed in future to ensure the management of existing target site mutations and possible upcoming additional mutations derived from selective pressure. Based on trifludimoxazin, we could show how to use binding groups to a β‐wall amino acid residue, which could be used in future to adapt further inhibitors in the PPO active site. However, technologies like DNA‐encoded library screening offer further ways to screen billions of molecules to find new herbicides. Although the target site resistance profile was assessed, we cannot exclude at this stage that nontarget site mechanisms might reduce the efficacy of PPO‐inhibiting herbicides, including trifludimoxazin.

## CONFLICTS OF INTEREST

Authors affiliated with BASF contributed to the planning and implementation of research activities. All other authors declare no conflict of interest.

## Data Availability

The data that support the findings of this study are available from the corresponding author upon reasonable request.
